# Predictive Utility of the HALP and Modified HALP Score for the Assessment of Operative Complications in Patients Undergoing Laparoscopic Cholecystectomy for Acute Cholecystitis

**DOI:** 10.3390/diagnostics15020152

**Published:** 2025-01-10

**Authors:** Yasemin Keskin, Hakan Sevinç, Selçuk Mevlüt Hazinedaroğlu, Şevket Barış Morkavuk, Şiyar Ersöz

**Affiliations:** 1 Department of General Surgery, Dr. Abdurrahman Yurtaslan Ankara Oncology Training and Research Hospital, Ankara 06200, Turkey; konuk.yasemin@gmail.com; 2 Department of General Surgery, Ankara University School of Medicine, Ankara 06200, Turkey; hakansevinc15@gmail.com (H.S.); selcukhazinedaroglu@gmail.com (S.M.H.); 3 Department of Surgical Oncology, Health Sciences University Gülhane Training and Research Hospital, Ankara 06018, Turkey; drsbmor@yahoo.com

**Keywords:** HALP score, modified HALP score, acute cholecystitis, complications, prognosis

## Abstract

**Background and Objectives**: The aim of the present study was to calculate HALP and modified HALP scores for patients diagnosed with acute cholecystitis (AC) and to determine the predictive utility of these scores for surgical timing and morbidity in patients who underwent surgery for AC. **Materials and Methods**: This study included data from 641 patients who underwent surgery for AC between January 2010 and May 2023. The HALP score was calculated using the formula hemoglobin (g/L) × albumin (g/L) × lymphocyte (10^9^/L)/platelets (10^9^/L). The modified HALP score was calculated using the formula hemoglobin (g/L) × albumin (g/L) × lymphocyte (10^9^/L) × platelets (10^9^/L). **Results**: The mean HALP score was 46.81, and the mean modified HALP score was 2,758,401.21. Laparoscopic cholecystectomy (LC) was successfully completed in 582 (90.8%) patients. When examining the relationship between HALP and modified HALP scores and the procedure type, a statistically significant difference was found in the distribution of median HALP and modified HALP scores between the LC and laparoscopic and open cholecystectomy (LTOC) groups. For patients with a HALP score < 34.43 and modified HALP score < 2,077,019, the likelihood of conversion to open surgery increased, with a sensitivity of 65.5% vs. 58.8% and a specificity of 66.1% vs. 59.3%. In patients who underwent surgery, there was a significant difference in the LTOC between the HALP score and modified HALP score cut-off groups (*p* < 0.001 and, *p* = 0.007). **Conclusions**: Evaluation of the HALP score is a promising and valuable assessment method for designing appropriate treatment and management strategies for patients with AC.

## 1. Introduction

Cholelithiasis is a common condition among Western societies, although the majority of patients are asymptomatic. It is estimated that 2–4% of asymptomatic patients become symptomatic within 1 year of diagnosis, while two-thirds of these patients are expected to remain asymptomatic. In asymptomatic patients, the probability of developing acute cholecystitis is 0.3%, the risk of obstructive jaundice is 0.2%, and the risk of acute pancreatitis ranges from 0.04% to 1.5% [1]. However, a significant increase in the incidence of complications has been observed, particularly in the pediatric patient group, and the most appropriate approach for the treatment of choledocholithiasis in this group remains unclear [2].

Acute cholecystitis (AC), the most common complication of cholelithiasis characterized by inflammation of the gallbladder, occurs in 90–95% of cases due to cystic duct obstruction by a gallstone and represents the initial clinical manifestation in 10–15% of patients [3]. Laparoscopic cholecystectomy (LC) is the standard procedure for patients undergoing surgery for gallstone(s). However, due to the inflammatory changes during the AC process, LC is not always feasible, and sometimes a need for laparoscopic to open cholecystectomy (LTOC) arises. Therefore, uncertainties regarding the optimal timing of AC surgery remain a current issue.

Immune suppression occurs during chronic inflammation and nutritional deficiency. Therefore, conditions accompanied by inflammation, such as AC, increase the risk for morbidity and mortality. The Hemoglobin, Albumin, Lymphocyte, and Platelet (HALP) score, developed by Chen et al. [4] and recently applied, reflect patient systemic inflammation and nutritional status.

It has been shown through studies that the HALP score can be an important prognostic marker, particularly in terms of survival, for various types of cancer [5,6,7,8]. This is thought to be due to hemoglobin, albumin, lymphocyte, and platelet levels being indicators of immune and nutritional status, each based on distinct underlying mechanisms. As superior indicators of immune–nutritional status, lymphocyte, hemoglobin, and albumin values are included in the numerator of the score, while platelets, as markers of disease, are included in the denominator [8].

In the context of inflammatory disease conditions, it was observed that the same parameters could also serve as indicators of inflammation, leading to the idea that the HALP score might be applicable in the evaluation of inflammatory diseases. However, a recent Letter to Editor noted that the increase in platelet count is an indicator of poor prognosis in malignant cases, which is why it is included in the denominator of the calculation formula. They argued that since platelets have a positive impact as a marker in the evaluation of benign conditions, they should be included in the numerator, leading to the development of the modified HALP score [9].

Because the HALP score is a newly developed scoring system, a clinically significant universal threshold value has not yet been determined, the formulation for benign/malignant diseases has not been clarified, and studies investigating to the HALP score remain ongoing. The aim of the present study, therefore, was to calculate HALP and modified HALP scores for patients diagnosed with AC and to determine the predictive utility of these scores for surgical timing and morbidity in patients who underwent surgery for AC.

## 2. Materials and Methods

### 2.1. Patient Selection

This study included data from 641 patients who underwent surgery for AC at Ankara University Faculty of Medicine Hospital (Ankara, Turkey) between January 2010 and May 2023. The study was accepted by the Ankara University Human Research Ethics Committee with the decision number İ07-493-23 on 7 August 2023.

The inclusion criteria were as follows: age ≥18 years; presence of AC associated with radiologically confirmed gallstone(s), and cholecystectomy performed in the presence of a diagnosis and clinical picture of AC.

Those with an age of 18 years, a presence of preoperative interventional procedure due to AC, chronic cholecystitis, an absence of a radiological diagnosis of gallstone(s), and a diagnosis of malignancy based on pathology results were excluded from the study.

### 2.2. Data Collection

Patient medical records from the Ankara University Faculty of Medicine database were retrieved and retrospectively reviewed. Patient demographic characteristics (age, sex), presence of comorbidities, American Society of Anesthesiologists (ASA) score, history of abdominal surgery, history of biliary colic/previous cholecystitis attacks, biochemical results of the current hospitalization, open/laparoscopic surgical status, and whether or not laparoscopic surgeries were converted to open surgery were determined.

### 2.3. Study Design

This study aimed to demonstrate the positive predictive utility of HALP and modified HALP scores as markers for determining surgical management and secondary morbidity in patients undergoing surgery for AC.

The primary endpoint of the study was to determine the utility of HALP and modified HALP scores for surgical morbidity in patients who underwent surgery for AC. The secondary endpoint was to compare the effectiveness of HALP and modified HALP scores as markers of inflammatory conditions.

No preoperative interventions that could influence inflammatory markers were performed on any patient in the study, and all patients were treated with the same analgesia and antibiotic therapy.

A validation cohort for the marker evaluated in the study has not been established, despite the recognized critical importance of such an analysis. However, given that the utilization of HALP score is still in its early stages and predominantly focuses on malignancies, this study primarily aims to highlight its potential applicability in the context of benign diseases.

### 2.4. HALP Score

The HALP score is an immuno-nutritional biomarker that incorporates values for hemoglobin (g/L), albumin (g/L), lymphocyte (10^9^/L), and platelets (10^9^/L), and is calculated using the following equation:

HALP score = hemoglobin (g/L) 0078 albumin (g/L) × lymphocyte (10^9^/L) / platelets (10^9^/L).

### 2.5. Modified HALP Score

The modified HALP score was calculated using the following equation:

Modified HALP score = hemoglobin (g/L) × albumin (g/L) × lymphocyte (10⁹/L) × platelets (10⁹/L).

### 2.6. Statistical Analysis

Statistical analysis and calculations were performed using IBM SPSS Statistics for Windows version 22.0 (IBM Corporation, Armonk, NY, USA). The normality of the data in the study was assessed through histogram analysis. The Pearson chi-square test and Fisher’s exact test were used to evaluate the nominal data of the groups, while Student’s *t*-test was used for the analysis of parametric data, and the Mann–Whitney U test was used for the analysis of nonparametric data. The cut-off sensitivity and specificity values were calculated using receiver operating characteristic (ROC) curve analysis. Values with *p* < 0.005 were considered statistically significant.

## 3. Results

This retrospective study included data from 641 patients (242 [37.8%] female, 399 [62.2%] male) with a mean (±standard deviation [SD]) age of 56.65 ± 15.88 years (range, 18 to 105 years). According to the ASA scoring system, 54.1%, 40.4%, 5% and 0.5% of patients were classified as ASA 1, ASA 2, ASA 3, and ASA 4, respectively. Of the 641 patients, 43.7% had comorbidities, whereas the remainder had no history of additional disease(s). Patient medical histories included the following: abdominal surgery (*n* = 37 [5.8%]); biliary colic (*n* = 499 [77.8%]); and AC attacks (*n* = 319 [49.8%]. Patient demographic characteristics are summarized in Table 1.

On patient admission, hemogram and biochemical analyses provided the requisite data to calculate the HALP and modified HALP scores. The mean HALP score was 46.81 ± 62.55, and the mean modified HALP score was 2,758,401.21 ± 2,090,392.08 SD (Table 2).

All patients were scheduled for LC due to AC. Laparoscopic surgery was successfully completed in 582 of 641 (90.8%) patients. However, LC was unsuccessful in 59 (9.2%), and the procedure was converted to LTOC.

HALP and modified HALP scores calculated in this study were subjected to homogeneity and distribution tests. Neither score exhibited a homogeneous distribution and was nonparametric. When examining the relationship between HALP and modified HALP scores and procedure type, a statistically significant difference was found in the distribution of median HALP scores between the LC and LTOC groups. The median HALP score were 42.73 in the LC group and 27.6 in the LTOC group (*p* < 0.001). Similarly, the distribution of the modified HALP scores between the 2 groups was statistically significant. The median modified HALP scores were 2,442,517 and 1,966,272 in the LC and LTOC groups, respectively (*p* = 0.001) (Table 3).

To validate the study hypothesis, a univariate regression test was performed using HALP and modified HALP scores. The model accuracy rate for both parameters was 90.8%. A negative relationship was found between an increase in HALP score and the likelihood of conversion to open surgery. A decrease in HALP score increased the likelihood of conversion to open surgery by a factor of 0.970 (Table 4).

**Figure 1:**
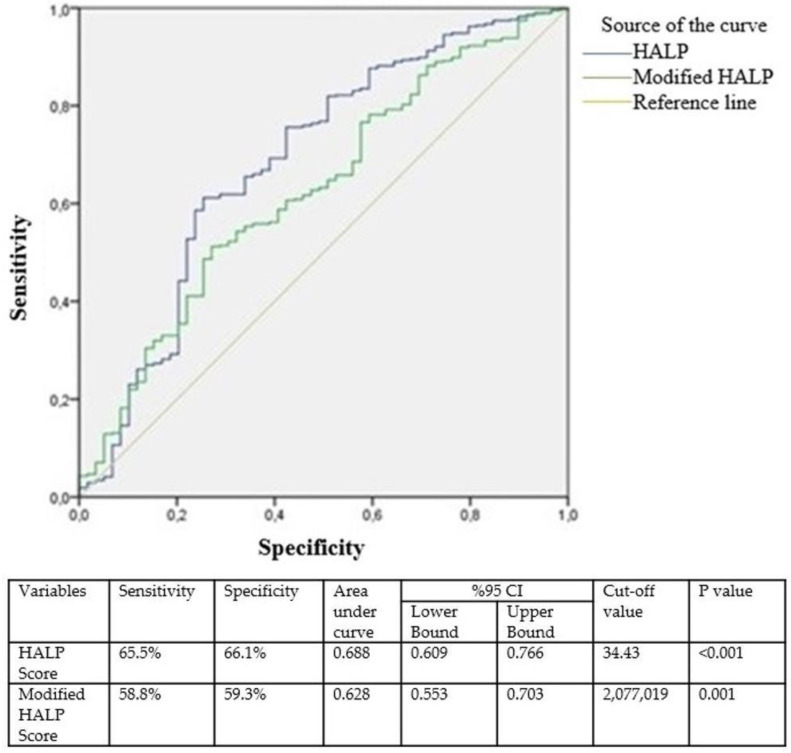
HALP score and modified HALP score ROC curve analysis for laparoscopic to conventional open surgery. Cl: confidence interval.

Given the significant relationship between the HALP score, modified HALP score, and procedure type (i.e., LC and LTOC), ROC curve analysis was performed. For patients with a HALP score < 34.43, the likelihood of conversion to open surgery increased, with a sensitivity of 65.5% and a specificity of 66.1%. Similarly, for patients with a modified HALP score < 2,077,019, the likelihood of conversion to open surgery increased, with a sensitivity of 58.8% and a specificity of 59.3% (Figure 1).

Patients were re-grouped according to cut-off values determined using ROC analysis for the HALP and modified HALP scoring systems. A total of 240 patients had a HALP score below the cut-off value and 401 a HALP score above it. For the modified HALP score, 275 patients were below the cut-off value and 366 were above the cut-off value. Patients were reclassified based on their demographic and clinicopathological data according to cut-off values for both groups.

When examining the distribution of patient demographic characteristics across the cut-off groups for both the HALP and modified HALP scores, a significant difference was found only in mean age. In the group with a low HALP score cut-off, the mean age was 59.19 years compared with 55.75 years in the high-score group (*p* = 0.007). Similarly, in the group with a low modified HALP score cut-off, the mean age was 59.49 years compared with 55.19 years in the high-score group (*p* = 0.001). There was no significant difference in sex distribution between the groups defined by the HALP or modified HALP score cut-offs. While the presence of known comorbidities did not exhibit a significant difference across the HALP score cut-off groups (*p* = 0.978), there was a significant difference in distribution across the modified HALP score cut-off groups (*p* < 0.001).

Regarding patient medical histories, the presence of biliary colic did not exhibit significant differences between the HALP and modified HALP score cut-off groups (*p* = 0.719 and, *p* = 0.086, respectively). There was no significant difference in a history of AC among the HALP score cut-off groups. However, regarding the modified HALP score, the presence of a history of AC exhibited a significant difference across the cut-off groups (*p* = 0.006).

In patients admitted due to AC, C-reactive protein (CRP) levels measured at the time of admission exhibited significant differences across both the HALP and modified HALP score cut-off groups (*p* < 0.001 and, *p* = 0.001, respectively). Regarding liver function tests (LFTs), when examining both groups according to their cut-off values, significant differences were found in aspartate aminotransferase (AST), gamma-glutamyl transferase (GGT), and alkaline phosphatase (ALP) levels. Alanine aminotransferase (ALT) exhibited statistical significance only in relation to the modified HALP score cut-off values (Table 5).

In patients who underwent surgery for AC, there was a significant difference in the conversion to open cholecystectomy (i.e., LTOC) between the HALP score and modified HALP score cut-off groups (*p* < 0.001 and, *p* = 0.007, respectively)

## 4. Discussion

LC is the standard procedure for patients undergoing surgery for gallstone(s). In AC, especially in cases of gangrenous and emphysematous cholecystitis, concerns have been raised about bile duct injuries due to local inflammation, increased blood loss, prolonged procedure, and increased morbidity and mortality compared with LC. However, with increased surgical experience and technological advances over the years, concerns regarding LC in patients with AC have diminished [3]. In a meta-analysis including patients who underwent early or delayed LC after AC between 2004 and 2015, Song et al. [10] found no significant differences in mortality, bile duct injury, bile leakage, overall complications, or conversion rates between the two groups. However, early LC treatment resulted in significant reductions in wound infections, hospital stays, operative duration, and improvements in quality of life compared with delayed treatment. According to the 2020 World Society of Emergency Surgery Guidelines, early LC (within the first 7 days of hospital admission) is recommended for the treatment of AC when there is access to an experienced surgeon. In cases for which an appropriate center or experienced surgeon is not available, delaying LC (between 6 weeks and 3 months) is recommended [3].

Despite numerous randomized controlled trials supporting the safety of LC in the treatment of AC, concerns have been raised by the complicated intra-abdominal infections worldwide observational study (CIAOW), a recent global study investigating intra-abdominal infections. This study involved 68 different centers over a period of 6 months and found that 48.7% of patients with AC underwent surgical treatment ending in open cholecystectomy [11]. In a meta-analysis conducted by Magnano San Lio et al. [12], the relationship between LC and conversion to open cholecystectomy (LTOC) in AC patients was examined, and significant results were found in the five studies included in this meta-analysis. The combined statistical evaluation of these studies determined the conversion rate to open cholecystectomy in AC patients to be 5.5%. In this study, the LTOC rate was found to be 9.2%. The noticeable decline in LTOC rates since 2014 highlights that laparoscopic cholecystectomy has become a promising option. It is believed that advancements in laparoscopic surgical technology and the increase in surgical experience have played a role in this process.

The treatment of AC typically involves cholecystectomy, with LC being the preferred treatment technique. Although LC is typically regarded to be safe, it has a mortality rate of 0.1–1% and bile duct injury rates of 0.2–1.5% [13]. In cases of AC, major complications, such as myocardial infarction, heart failure, renal failure, and pulmonary embolism, have been reported in approximately 6–9% of patients [14]. Although LC is regarded as the standard treatment for patients with AC, non-surgical approaches are recommended for individuals classified as unfit for surgery. However, the definition and characterization of the “unfit for surgery” patient profile remain ambiguous and subject to ongoing debate. Hence, identifying patients who are at high risk for complications, morbidity, and mortality before surgery is crucial. These patients should be referred to higher-level centers or experienced surgeons. Determining these risks, such as demographic characteristics of the patients, pre-existing gallbladder-related symptoms, and biochemical and radiological features at the time of admission, can help in determine the most beneficial treatment approach for patients.

In patients with AC, various grading systems have been previously explored to define disease severity and predict postoperative complications and mortality [15,16]. However, the heterogeneity of the AC patient profile has been highlighted as a significant barrier to the development of a standardized predictive algorithm. This study aims to address this challenge by minimizing the variability associated with patient, disease, and surgeon-related factors and developing a predictive algorithm grounded in objective data with established efficacy in assessing inflammation.

In this study, HALP and modified HALP scores were used to identify risk factors in patients with AC. HALP includes parameters routinely tested in patients followed up for AC, making it simple and cost-effective to calculate. It evaluates both the immune system and the nutritional status of patients. Studies have indicated that the HALP score has prognostic value for various cancers, including gastrointestinal and genitourinary cancers [4,17,18,19,20,21]. In a study involving patients diagnosed with ileus, Bildik et al. [22] reported that patients with low HALP scores had a higher risk for mortality and need for surgical treatment. Antar et al. [23] focused on patients with asthma, which is a benign condition. The authors found lower HALP scores in patients diagnosed with asthma; however, they concluded that this finding was not associated with the risk for asthma. The study also examined patients with arthritis, including those with rheumatoid arthritis or osteoarthritis, who had lower HALP scores than those in the non-inflammatory group. Increased platelet-to-lymphocyte ratios and decreased hemoglobin levels were observed in patients with thyroiditis. However, there was no statistically significant relationship between HALP scores and thyroiditis. This observation may be related to the variations in the etiology of thyroiditis. In addition, no specific study has evaluated the prognostic significance of HALP score in patients diagnosed with AC.

Hemoglobin levels serve as indicators of inflammation-aggravated anemia when examining the components of the HALP score. Albumin, which is synthesized by the liver, is a negative acute-phase reactant used to assess nutritional status. Hypoalbuminemia can result from inadequate nutrition, hypercatabolism, systemic inflammation, or an increased release of cytokines [24]. Thrombocytopenia observed during inflammation results from thrombocytes aggregating at the site of inflammation, adhering to leukocyte surfaces, and forming aggregates, which leads to a decrease in their circulating numbers. Therefore, the platelet count is a parameter used to grade organ failure in sepsis.

In a Letter to Editor, Dai et al. [9] noted that elevated platelet counts were associated with cancer progression and metastasis. Therefore, high platelet levels in patients with cancer are indicative of poor prognosis and are included in the calculation of the HALP score through division. Additionally, the authors reported that, unlike patients with cancer, those with infections tend to exhibit decreased platelet counts, which is correlated with disease severity. Therefore, in non-cancerous diseases, multiplication rather than division of platelet counts is recommended when calculating the score. Based on current knowledge, the suggested approach is to calculate the modified HALP score.

This study aims to guide the development of a treatment algorithm for AC by comparing HALP and modified HALP scores in the context of LC and LTOC. It is recommended that conservative treatments be prioritized over surgical interventions in patients with HALP scores below 34.43 and modified HALP scores below 2,077,019. Patients falling below these thresholds demonstrated increased rates of LTOC and were identified as being at higher risk for surgical complications.

Alshuweishi et al. [25] noted that older individuals (≥65 years of age) tended to exhibit higher HALP scores. This association is attributed to the increased inflammation with aging, which subsequently leads to elevated lymphocyte and platelet counts, thereby increasing the HALP score. However, in the current study, older patients were found in the groups below the HALP and modified HALP score cut-off values. Similarly to our study, Antar et al. [23] conducted a cross-sectional study in the United States involving 8245 patients and reported an inverse relationship between HALP score and age. The authors demonstrated that for each year of increase in age, there was a decrease of 0.86 in the HALP score. They attributed this trend to the age-related decline in hemoglobin and albumin levels. Based on study data, considering the significant association between HALP score and LTOC in patients with AC, the choice of surgical approach in patients >60 years of age should be thoroughly evaluated, accounting for all aspects, including surgical morbidity. Delayed surgical treatment should be considered based on surgical morbidity considerations.

Male sex was associated with an increased risk for AC-related complications (15% risk, 95% confidence interval [CI]) and a higher likelihood of conversion from LTOC (48.5% risk, 95% CI). The exact cause of this increase is not definitively understood; however, it is believed to be related to increased muscle mass, especially in the trunk, and visceral adiposity, which is more common among males [3]. Based on these data, the relationship between sex and HALP score was investigated. A large nationwide study examining the association between dyslipidemia and HALP scores reported that males had HALP scores that were approximately 10% higher than those in females. This finding has been attributed to the differences in laboratory reference values between males and females [25]. However, in the current study, no significant differences were found in terms of HALP and modified HALP scores based on sex. This suggests that determining the surgical management approach for patients with AC based on sex may not be suitable.

In patients with AC, changes in LFTs are observed due to acute inflammation of the gallbladder and bile ducts rather than bile duct obstruction. Approximately 15–50% of patients with AC exhibit elevated LFT values without obstruction of the main bile ducts [3]. Based on this perspective, the current study compared the relationships among LFT values, HALP and modified HALP scores among patients. When examining LFT values according to the cut-off values of both groups, significant differences were found for AST, GGT, and ALP, whereas ALT was significant only for the modified HALP score cut-off values. Based on these findings, it would be appropriate to consider delaying cholecystectomy in patients with AC with elevated LFT values, and accounting for morbidity associated with early surgical treatment.

Based on the current data and a literature review, no clear superiority has been observed between HALP and modified HALP scores in patients with AC. As reported by Dai et al. [9], the necessity of calculating the modified HALP score under inflammatory conditions, particularly in patients with a history of AC, was found to be significant. Evaluation of patient admission tests and surgical management have shown that calculating the HALP score at admission is sufficient. An increased likelihood of requiring LTOC was observed in patients with HALP scores below the cut-off value. Therefore, calculating the HALP score in patients scheduled for surgery for AC may be meaningful in terms of morbidity, suggesting that patients may benefit from delaying cholecystectomy, if necessary.

### Limitations

Given the limited number of studies investigating the HALP score and its association with malignancies, there is a need for comprehensive and prospective studies to substantiate the current findings.

The retrospective design of the present study precluded the use of additional inflammation assessment scales to evaluate the inflammatory status of patients. Prospective studies comparing the HALP score with other markers would be beneficial for enhancing the accuracy of the current findings. Additionally, a larger population-based and/or multicenter prospective study could provide more robust data regarding the sensitivity and specificity levels of the HALP score.

A validation cohort for the marker evaluated in this study has not been established, despite the recognized critical importance of such an analysis. However, given that the utilization of HALP score is still in its early stages and predominantly focused on malignancies, this study primarily aims to highlight its potential applicability in the context of benign diseases.

## 5. Conclusions

Identifying patients who are at high risk for complications and mortality before surgery is crucial for planning surgical procedures and referring patients to advanced centers and specialist physicians. Assessment of these risks and potential outcomes can guide decisions regarding surgical intervention and conservative management. In this context, evaluation of the HALP score is a promising and valuable assessment method for designing appropriate treatment and management strategies.

## Figures and Tables

**Table 1:** The demographic characteristics of the patients.

Age, year, mean ± SD	56.65 ± 15.88 (18–105)
Gender: *n* (%)	
Male	242 (37.8%)
Female	399 (62.2%)
ASA: *n* (%)	
Asa 1	347 (54.1%)
Asa 2	259 (40.4%)
Asa 3	32 (5%)
Asa 4	3 (0.5%)
Presence of comorbidities: *n* (%)	
Absent	361 (56.3%)
Present	280 (43.7%)
History of previous abdominal surgery: *n* (%)	
Absent	604 (94.2%)
Present	37 (5.8%)
History of acute cholecystitis: *n* (%)	
Absent	322 (50.2%)
Present	319 (49.8%)
History of biliary colic pain: *n* (%)	
Absent	142 (22.2%)
Present	499 (77.8%)

ASA: American Society of Anesthesiologists score.

**Table 2:** Laboratory findings and HALP—modified HALP scores of patients.

CRP, mg/dL, mean ± SD	51.80 ± 79.17 (0.1–500)
ALT, U/L, mean ± SD	50.41 ± 88.95 (2–1207)
AST, U/L, mean ± SD	43.50 ± 80.64 (0.2–1300)
GGT, U/L, mean ± SD	89.17 ± 159.59 (7–1658)
ALP, IU/I, mean ± SD	94.11 ± 84.23 (5–937)
Hemoglobin, g/dL, mean ± SD, range	13.17 ± 17.04 (7.4–17.7)
Albumin, g/dL, mean ± SD, range	3.88 ± 5.90 (1.6–5.9)
Lymphocyte, 10^3^/μL, mean ± SD, range	2.10 ± 1.89 (0.2–36.7)
Platelet, 10^3^/μL, mean ± SD, range	253.11 ± 73.48 (91–637)
HALP Score, mean ± SD, range	46.81 ± 62.55 (03.1401.4)
Modified HALP Score, mean ± SD, range	2,758,401.21 ± 2,090,392.08 (123,667–21,896,138)

CRP: C-reactive protein; ALT: alanine aminotransferase; AST: aspartate aminotransferase; GGT: gamma-glutamyl transferase; ALP: alkaline phosphatase; SD: standard deviation.

**Table 3:** Association between operation type and HALP—modified HALP scores.

Systemic Inflammation Scoring Systems	No. of Patients (%)		*p* Value
	Laparoscopic Cholrcystectomy (582 Patients 90.8%)	Conventional Open Cholecystectomy (59 Patients 9.2%)	
HALP Score	42.73 (3.1–1401.4)	27.6 (3.2–105.9)	*p* < 0.001 ^U^
Modified HALP Score	2,442,517 (123,667–21,896,138)	1,966,272 (220,080–6,279,582)	*p* = 0.001 ^U^

^U^: Mann–Whitney U test.

**Table 4:** Univariate regression analysis of systemic inflammation scoring systems for operation type.

Dependent Variable	Overall Percentage Correct	*p* Value
HALP Score	90.8%	<0.001
Modified HALP Score	90.8%	0.004

**Table 5:** Relationship between HALP score and modified HALP score groups with clinico-pathological factors.

Variables	HALP Score			Modified HALP Score		
	Low Cut-Off Value 240 Patients	High Cut-Off Value 401 Patients	*p* Value	Low Cut-Off Value 275 Patients	High Cut-Off Value 366 Patients	*p* Value
Age, year, mean ± SD	59.19 ± 16.08	55.75 ± 15.36	0.007 ^T^	59.49 ± 16.08	55.19 ± 15.19	0.001 ^T^
Gender: *n*						
Male	90	152	0.918 ^×2^	108	134	*p* = 0.549 ^×2^
Female	150	249		167	232	
History of acute cholecystitis: *n* (%)						
Absent	115	207	0.364 ^×2^	121	201	0.006 ^×2^
Present	125	194		154	165	
History of biliary colic pain: *n* (%)						
Absent	55	87	0.719	52	90	0.086 ^×2^
Present	185	314		223	276	
Presence of comorbidities: *n* (%)						
Absent	135	226	0.978 ^×2^	133	228	<0.001 ^×2^
Present	105	175		142	138	
CRP, mg/dL, median, range	44.45(0.3–456)	7 (0.1–500)	<0.001 ^U^	13 (0.3–456)	10.25 (0.2–500)	0.001 ^U^
ALT, U/L, median, range	24.5 (2–1207)	25 (6–743)	0.766	26 (2–1207)	23 (6–743)	0.008 ^U^
AST, U/L, median, range	25 (0.2–1300)	22 (7–350)	<0.001 ^U^	26 (0.2–1300)	21 (9–415)	<0.001 ^U^
GGT, U/L, median, range	37.5 (7–1658)	29 (7–1422)	0.025 ^U^	35 (7–901)	28.5 (7–1658)	0.004 ^U^
ALP, IU/I, median, range	81.5 (5–937)	70 (10–396)	<0.001 ^U^	76 (5–937)	71 (10–827)	0.013 ^U^
Laparoscopic to open cholecystectomy: *n* (%)			<0.001 ^×2^			0.007 ^×2^
Absent	201	381		240	342	
Present	39	20		35	24	

CRP: C-reactive protein; ALT: alanine aminotransferase; AST: aspartate aminotransferase; GGT: gamma-glutamyl transferase; ALP: alkaline phosphatase; ^T^: Student’s *t*-test; ^×2^: Chi-square test; ^U^: Mann–Whitney U test; SD: standard deviation.

## Data Availability

Data available on request due to restrictions.
